# Bacterial Diversity Evolution in Maya Plaster and Stone Following a Bio-Conservation Treatment

**DOI:** 10.3389/fmicb.2020.599144

**Published:** 2020-11-09

**Authors:** Fadwa Jroundi, Kerstin Elert, Encarnación Ruiz-Agudo, María Teresa Gonzalez-Muñoz, Carlos Rodriguez-Navarro

**Affiliations:** ^1^Department of Microbiology, University of Granada, Granada, Spain; ^2^Department of Mineralogy and Petrology, University of Granada, Granada, Spain

**Keywords:** Maya area, bacterial diversity, bioconsolidation treatment, nutritional medium, carbonatogenic bacteria, tuff stone, plasters

## Abstract

To overcome the limitations of traditional conservation treatments used for protection and consolidation of stone and lime mortars and plasters, mostly based on polymers or alkoxysilanes, a novel treatment based on the activation of indigenous carbonatogenic bacteria has been recently proposed and applied both in the laboratory and *in situ*. Despite very positive results, little is known regarding its effect on the evolution of the indigenous bacterial communities, specially under hot and humid tropical conditions where proliferation of microorganisms is favored, as it is the case of the Maya area. Here, we studied changes in bacterial diversity of severely degraded tuff stone and lime plaster at the archeological Maya site of Copan (Honduras) after treatment with the patented sterile M-3P nutritional solution. High-throughput sequencing by Illumina MiSeq technology shows significant changes in the bacterial population of the treated stones, enhancing the development of *Arthrobacter*, *Micrococcaceae*, *Nocardioides*, *Fictibacillus*, and *Streptomyces*, and, in one case, *Rubrobacter* (carved stone blocks at Structure 18). In the lime plaster, *Arthrobacter*, *Fictibacillus*, *Bacillus*, *Agrococcus*, and *Microbacterium* dominated after treatment. Most of these detected genera have been shown to promote calcium carbonate biomineralization, thus implying that the novel bio-conservation treatment would be effective. Remarkably, the treatment induced the reduction or complete disappearance of deleterious acid-producing bacteria such as *Marmoricola* or the phylum *Acidobacteria*. The outcome of this study demonstrates that such a bio-conservation treatment can safely and effectively be applied on temples, sculptures and stuccos of the Maya area and, likely, in other hot and humid environments.

## Introduction

Stone and lime plaster deterioration is one of the most serious problems affecting historical structures and sculptures all over the world (Warscheid and Braams, 2000; Gil et al., 2015). Deterioration is due to physical, chemical, and biological weathering phenomena, acting alone or in combination, which frequently result in irreparable loss of priceless artworks. In aggressive tropical environments, characterized by heavy rainfalls, high temperatures and high relative humidity, typical for most Maya archeological sites in Mesoamerica, these phenomena are particularly harmful and enhance biodeterioration related to the impact of different (micro)organisms (Videla et al., 2000; Caneva et al., 2005). Biodeterioration intensity depends on the material, possible previous interventions, and environmental conditions that determine the extent and composition of the microbial communities colonizing the cultural artifacts (Valentín, 2010).

Microorganisms are able to cause several types of damage on monument surfaces, including biophysical, biochemical, and aesthetic biodeterioration, which may occur simultaneously or separately (see reviews by Warscheid and Braams, 2000; Valentín, 2010; Gil et al., 2015; Mihajlovski et al., 2017; Sterflinger et al., 2018). Although some microorganisms can cause biodeterioration, many of them can offer a very effective solution for the conservation of deteriorated historical sculpture and monuments.

The conservation of such historic and culturally important artworks typically involves the application of consolidating agents that in many cases do not provide long-lasting efficacy and induce further damage due to pore blocking as well as severe aesthetic alterations (Giorgi et al., 2010; Sassoni et al., 2011). Bacterial bioconsolidation has emerged in recent decades as an alternative to non-effective traditional consolidants. This environmentally friendly conservation treatment involves the consolidation of stone and plaster through bacterially induced calcium carbonate biomineralization (González-Muñoz et al., 2008). Bacterial biomineralization is a widespread phenomenon reported for many natural environments such as soils and caves (Boquet et al., 1973; Wright and Oren, 2005). This strategy is effective for the protection of stone or plaster artworks because it can form exceptionally strong hybrid organic-inorganic carbonate cements (Jroundi et al., 2017). One particularly effective strategy involves the application of a nutritional solution (M-3P) that selectively activates indigenous carbonatogenic bacteria present in the treated substrate (Rodriguez-Navarro et al., 2003, 2012, 2015; Ettenauer et al., 2011; Jroundi et al., 2012, 2017). In fact, the application of the patented M-3P nutritional solution containing amino acids (Bacto Casitone) and calcium, promotes the growth of the indigenous chemoorganotrophic bacteria that are able to use amino acids as a source of carbon, nitrogen, and energy. The bacterial metabolic activity involves the oxidative deamination of amino acids and the subsequent release of ammonia, which leads to the formation of NH_4_^+^ and OH^–^ ions and induces an increase in pH. Under these alkaline conditions, dissolved CO_2_ (both atmospheric and metabolically derived CO_2_) transform into CO_3_^2–^ ions that react with Ca^2 +^ (present in the M-3P medium), leading to the precipitation of calcium carbonate, once a sufficient supersaturation is reached (Rodriguez-Navarro et al., 2003, 2015; Jroundi et al., 2012).

The first step to understand the relationship between microorganisms and environment, as well as its potential response during a bio-conservation treatment, is to identify the bacteria present in the cultural artwork. Next generation sequencing approaches have been developed to study the complexity of microbial communities in a wide range of environments, allowing in-depth studies of environmental samples (Marvasi et al., 2019). Such sequencing technologies, in addition to other molecular approaches, have also been applied to analyze the diversity, composition and distribution of microbial communities dwelling on different historical objects (Zimmermann et al., 2006; Piñar et al., 2015; Mihajlovski et al., 2017; Adamiak et al., 2018; Li et al., 2016, 2018), all of them mainly focusing on the biodeteriorating role of these dwelling microorganisms. However, monitoring the evolution of such microbial communities, before and after the application of conservation treatments, is rarely reported in the literature. Here, we apply DNA extraction, PCR amplification and high-throughput sequencing and use bioinformatics and statistical analysis to characterize and compare bacterial colonization on historic Maya mortar and stone before and after a bioconsolidation treatment applied in the laboratory and *in situ* at the archeological site of Copan. Up to now, this bacterial conservation treatment based on the application of a sterile nutritional solution (M-3P) has only been applied *in situ* on monuments located in temperate, moderately humid-dry European environments (e.g., Spain and Portugal) (Jroundi et al., 2012, 2017; Rodriguez-Navarro et al., 2015; Delgado-Rodrigues and Ferreira-Pinto, 2019). It is therefore unknown, whether such a treatment is effective and does not produce any deleterious side effects under extreme hot and humid environmental conditions typical for the Maya area, where the bacterial population is unknown and the potential for microbial biodeterioration is very high. The outcome of this study will be crucial for the evaluation of the effectiveness of bacteria-assisted consolidation treatments in tropical climates and lay the basis for the development of effective novel conservation strategies for the Maya area. The approach presented here will also be imperative for the understanding of natural biosystems and to determine the effect and efficiency of different conservation treatments (e.g., biocides and conventional organic consolidants, which could serve as a nutritional source for microorganisms). Furthermore, it could assist in the selection of appropriate measures (e.g., climate control) to limit/restrain the impact of deteriogenic communities on works of art, an important aspect in conservation and restoration practice. In summary, this study focuses on determining the impact of the biotreatment on the substrates’ bacterial diversity as a means to gauge the applicability of this environmentally friendly bioconsolidation approach in tropical regions. In a parallel study we are currently evaluating the effectiveness of the treatment applied on these Maya stones and plaster by analyzing the formation of bacterial calcium carbonates and their impact on the physical-mechanical properties of the treated substrates.

## Materials and Methods

### Site Description

**FIGURE 1:**
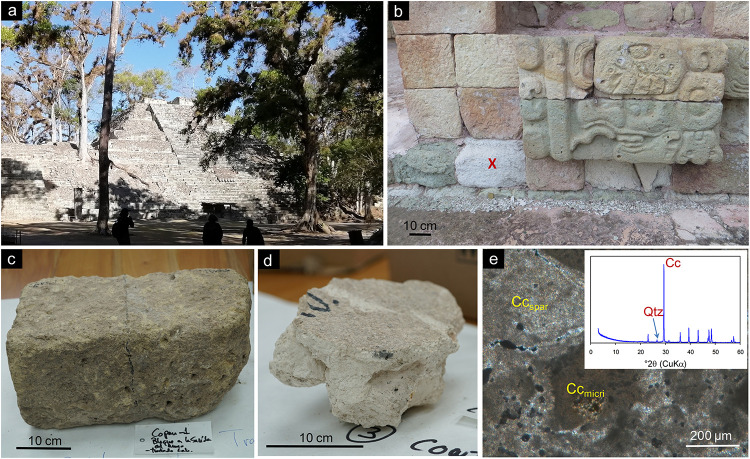
The Maya site of Copan: **(a)** general overview of Copan’s Acropolis; **(b)** stone blocks treated at Structure 18. The red x marks the treated stone block. Note the large amount of stone debris located at the bottom of the wall; **(c)** detail of the tuff stone treated at the LACEM laboratory; **(d)** picture of the plaster treated at the LACEM laboratory; **(e)** optical microscopy photomicrograph of the Maya plaster (crossed polarizer). Both micritic calcite (Cc_*micri*_) and sparitic-microsparitic calcite (Cc_*spar*_) are observed making up this (almost pure) lime plaster (no aggregate is visible in the images). The inset shows the X-ray diffraction pattern of the plaster. The main Bragg reflections of minerals present in the plaster are indicated (Cu Kα radiation). Cc, calcite; Qtz, quartz.

This study was performed at the Maya site of Copan, Honduras (Figure 1), one of the most important centers of the Mayan civilization, which has been excavated since the 19th century and was designated a World Heritage Site by UNESCO in 1980. This archeological site represents one of the most spectacular achievements of the Classic Maya period (AD 420–820), involving a number of architectural and sculptural monuments constructed and extensively carved using a local volcanic tuff stone, and decorated with lime plaster (Fash, 1991). It is composed of complex ruins with several secondary complexes surrounding them, including the well-known Hieroglyphic Stairway, displaying the longest known Classic Maya inscription (Fash et al., 1992; Webster, 1999). The remains are not only endangered by physico-chemical and biological deteriorating agents, but also by the continued erosive action of the Copan river, current land use, and several restoration/conservation interventions (performed during the 20th century) involving the use of non-compatible materials such as Portland cement (Doehne et al., 2005). Copan is situated on the southeastern frontier of the Lowland Maya culture area; what is today western Honduras (latitude, 14° 51′ 30″ N, longitude, 89° 9′ W) (Webster, 1999). The site is located at ∼700 m a.s.l., has an average annual *T* of ∼26°C and precipitation averaging ∼2000–3000 mm annually (McVey, 1998). It is therefore considered a hot and humid tropical environment.

### Bioconsolidation Treatment

For the testing of the bioconsolidation treatment based on the application of the patented M-3P nutritional solution, two types of substrates (tuff stone and lime plaster) were considered in this study:

(1) Stone block (sample CI) located in the base section of a vertical wall of a Late Classic Temple (Structure 18) made up of a buff-colored carved tuff stone, showing extensive deterioration and material loss due to scaling (Figure 1B). The treatment was applied *in situ* (see details below). The mineralogy and main weathering mechanisms of this volcanic tuff stone are reported in Doehne et al. (2005). The authors indicate that this rhyolitic tuff is relatively porous (∼20%) and made up of a devitrified silicate glass matrix (made up of zeolites and clay minerals), with abundant phenocrystals of quartz and feldspars. It typically displays extensive flaking and scaling, resulting in continuous loss of stone and surface reliefs. Lost material tends to accumulate at the bottom of exposed walls, as can be seen in Figure 1B. The ultimate cause of such an advanced weathering is not clear, although it has been claimed that microbial biodeterioration and humidity play an important role (Caneva et al., 2005; Doehne et al., 2005).
(2) Decontextualized stone block (sample CPN) originally located on the ground next to the Copan Acropolis (Figure 1C). The stone was partially covered by soil and plant leaves. This buff colored tuff stone block was treated *ex situ* at the *Laboratorio de Conservación de Escultura Maya* (LACEM; Laboratory for the Conservation of Maya Sculpture), which is part of the Copan Sculpture Museum (located within the site premises).
(3) Block of Late Classic lime plaster floor (sample MC) from Structure 12 (AD ∼800). After excavation, it was stored at the Copan Sculpture Museum storage area. This plaster (Figure 1D) was treated *ex situ* at the LACEM. Preliminary mineralogical and petrographic analyses show that it is made up of calcite (binder) with a minor amount (<5 wt%) of quartz (according to X-ray diffraction analysis performed on a X’Pert PRO, PANalytical diffractometer using a CuKα source, 3–60 °2θ explored range and 0.01 °2θ/min goniometer speed) (see inset in Figure 1E). Under the polarized light petrographic microscope (Jenapol V), it shows a compact but highly porous structure made up of micritic and microsparitic calcite crystals (Figure 1E). Basically, it is a pure lime plaster with almost no aggregate.

Note that this biotreatment application was a trial aimed at gauging its potential effectiveness under the particular exposure conditions in this tropical region. In order to minimize possible negative side-effects in case of treatment failure (i.e., possible activation of harmful bacteria leading to substrate acidification or discoloration), we have restricted the extent of its application for minimum impact. The extent of the testing and sampling was further restricted taking into account the value and uniqueness of this Maya site. Therefore, the three selected substrates were chosen considering their representativeness regarding the different materials and exposure conditions at the site. The first substrate (stone at Structure 18) is representative of the Copan tuff stones of different structures at the Copan Acropolis; the decontextualized stone block is representative of the excavated stone blocks and sculptures currently located at the Copan Sculpture Museum and storage area; and the plaster is representative of interior lime plaster floors and walls in structures at the Copan Acropolis, as well as plaster pieces located at the Copan Sculpture Museum and storage area.

The treatment application procedure is described in detail in Jroundi et al. (2017). Briefly, it involves the application of the sterile M-3P solution which contains 1% [wt./vol.] Bacto Casitone (a pancreatic digest of casein), 1% [wt./vol.] Ca(CH_3_-COO)_2_⋅4H_2_O (total calcium: 43.44 mM), 0.2% [wt./vol.] K_2_CO_3_⋅1/2H_2_O (total potassium: 35.6 mM; total carbonate: 17.8 mM), and 10 mM phosphate buffer in distilled water (pH = 8) (Rodriguez-Navarro et al., 2015). The nutritional solution was sprayed onto the stone/plaster blocks until saturation, twice a day for six consecutive days. Areas were protected from light exposure with cardboards during treatment. The treatment was performed during the dry season (February) with outdoors *T* ranging from 18 to 28°C and RH of ∼80 ± 5%. Note that the *T* and relative humidity (RH) within the LACEM during treatment was not controlled (∼20–26°C, ∼80 ± 5% RH), thus basically reflecting the outdoors conditions.

Both *in situ* and *ex situ* treatment applications where performed considering that future conservation interventions should be performed both *in situ*, on the different structures exposed outdoors at this Maya site (e.g., Hieroglyphic Stairway), as well as *ex situ* (indoors), on the vast collection of carved stone, sculptures, and plasters currently exhibited at the Copan Sculpture Museum as well as in the storage facilities. Once the treatment effectiveness is fully evaluated, a feasibility study for the scaling up of the treatment will be performed.

### DNA Extraction, PCR Amplification, and Sequencing

Genomic DNA was extracted from solid samples collected aseptically (using sterile tweezers, and sterile Eppendorf tubes) from all three samples, both before treatment application (CI_CONT, CPN_CONT, and MC_CONT: taken at time 0, before the application of M-3P nutritional solution) and 3 months after treatment application (CI_ TREATED, CPN_TREATED, and MC_TREATED). Three replicates of each sample were performed. After collection at the site, each sample/replicate (∼0.5 g) was aseptically placed in a 2-mL sterile screw-cap tube containing glass beads. One mL of lysis buffer (100 mM Tris-HCl [pH 8.0], 100 mM EDTA [pH 8.0], 100 mM NaCl, 1% polyvinylpyrrolidone [PVP], and 2% SDS), 24 μL freshly made lysozyme (10 mg/mL), and 2 μL proteinase K (20 mg/mL) were added to each tube. The tubes were vigorously shaken for 2 min in vortex followed by mechanical lysis of the cells performed twice using a FastPrep^®^ FP120 (at a speed of 5.5 m/s for 45 s) with intermittent cooling on ice for 5 min. The tubes were incubated at 37°C for 30 min and then at 60°C for another 30 min, and subsequently centrifuged at 14,000 × *g* for 5 min at room *T*. The upper (aqueous) phase was mixed by gently inverting with one volume of phenol:chloroform:isoamyl alcohol (25:24:1, pH 8), and then centrifuged at 1,500 × *g* for 10 min at 4°C. The upper (aqueous) phase was washed with one volume of phenol:chloroform (1:1) and centrifuged under the same conditions. DNA was precipitated by adding 1/10 volume of sodium acetate (3 M, pH 5) and one volume of isopropanol, incubating for 1 h at −80°C and centrifuging for 30 min at 5,000 × *g* at 4°C. The obtained DNA was dissolved in Tris (5 nM, pH 8.5)-TE buffer (10 mM Tris-HCl [pH 8.0] and 1 mM EDTA) previously heated at 65°C. Extracted DNAs were stored at −20°C until all sample extractions were completed. The DNA concentrations were determined on Qubit 3.0 Fluorometer using the dsDNA HS (high sensitivity) assay kit (Jiang et al., 2015).

High-throughput amplicon sequencing using 250 bp paired-end sequencing chemistry (MiSeq Illumina) was performed. Total DNA of each sample was amplified targeting the hypervariable V3-V4 regions by using the 16S rRNA gene primers 341F and 785R (Klindworth et al., 2013). Illumina libraries were constructed and sequenced at LGC Genomics^1^, GMBH, Berlin, Germany. The resulting DNA libraries were pooled equimolarly and barcoded to be sequenced in one single Illumina lane.

### Bioinformatics and Statistical Analysis

Demultiplexing of all libraries was carried out for each sequencing lane using the Illumina bcl2fastq. After quality controlling and combining using PandaSeq (Masella et al., 2012), paired-end reads were renamed using BESPOKE software (SeqSuite^2^) and then analyzed through the QIIME v1.8 pipeline (Caporaso et al., 2010). UCLUST and SILVA 119 QIIME 16S database were used to identify, classify and annotate operational taxonomic units (OTU). Explicet (2.10.5) was finally used to analyze clustered and annotated OTU. Rarefaction curves were determined using the Vegan v2.4-6 package in R v3.4.3 environment (data not shown).

Species richness was measured through the use of alpha-diversity metrics (Chao1, Shannon diversity index, and observed species) in Explicet and R softwares. Beta-diversity, the similarity between the identities of taxa, and their abundances in each sample were assessed using Bray-Curtis distances (weighted UniFrac distances) measured in QIIME and PAST3 v. 3.18 and the output was visualized by means of principal coordinate analysis (PCoA). A heatmap was constructed for the visualization of specific differences in community composition using the heatmap.2 function in the R gplots v2.11.0 package on log-normalized abundance data. At the genus level, the heatmap included only taxa at ≥1.5% relative abundance in all samples/replicates. Relative abundance graphs were constructed representing relative microbial abundance averages of three biological replicates. Additionally, similarity of percentages analysis (SIMPER) was performed using PAST3 software. Network analyses were conducted in the R environment using the VEGAN package and only strong Pearson’s correlations (ρ > 0.8 or ρ < −0.8) were considered. Network visualization and modularization were carried out on the interactive platform of Cytoscape (Shannon et al., 2003).

### Data Availability

All raw sequences used in this study are available in the sequence read archive (SRA) at NCBI database under the SRA accession number PRJNA650538.

## Results

### Richness and Distribution of the Bacterial Communities

A total of 910,782 bacterial 16S rRNA gene sequences were recovered for all samples. They were distributed in a mean of 63,420 for CI_CONT, 40,835 for CI_TREATED, 46,518 for CPN_CONT, 219,251 for CPN_TREATED, 4,990 for MC_CONT, and 46,108 for MC_TREATED. These sequences were used for community analyses by QIIME and OTUs were assigned by clustering sequences with over 97% sequence identity. A number of 1,165 OTUs were identified, indicating high microbial diversity.

Alpha-diversity analysis revealed no significant differences in bacterial richness of the studied samples regardless of the metric used (Table 1). Chao1 richness estimator and observed species index (Sobs) indicated high and comparable bacterial richness in the communities of both stone and plaster samples. As seen in Table 1, the samples have many sequences, which were also confirmed by Shannon, Simpson and the Goods coverage indexes. The samples displayed high richness and had similar alpha-diversities. According to Simpson index, all taxa were distributed equally in the CI bacterial communities after treatment with an average ranging from 0.53 to 0.56, whereas in the rest of the samples more than one taxon were dominating the communities with a Simpson index ranging from 0.89 to 0.98.

**TABLE 1:** Inference statistics at genus level of the different samples of the Maya archeological site.

SAMPLES	Taxa_richness	Simpson_1-D	Shannon_H	Pielou’s evenness	Fisher’s Alpha	ACE	CHAO1	Goods coverage index
CI_CONT	1561	0.94	3.77	0.51	291.81	137.3	139.1	0.999
CI_TREATED	496	0.54	1.8	0.29	79.8	105.9	107.4	0.999
CPN_CONT	2592	0.96	5.10	0.65	603.1	315.1	314.4	0.998
CPN_TREATED	4619	0.91	3.61	0.43	850.1	219.6	223.1	0.998
MC_CONT	239	0.98	4.65	0.85	47.9	85.3	85.9	0.998
MC_TREATED	1108	0.93	3.67	0.53	211.2	141	138.1	0.999

*The species richness (Taxa_richness, CHAO1, ACE), evenness (Pielou’s evenness, Simpson_1-D), and diversity (Shannon_H, Fisher’s alpha) indexes were calculated with a 3% distance cutoff. CI: Temple structure located outdoors; CPN: stone sample block located in the LACEM laboratory; and MC: lime plaster block located in the LACEM laboratory.*

**FIGURE 2:**
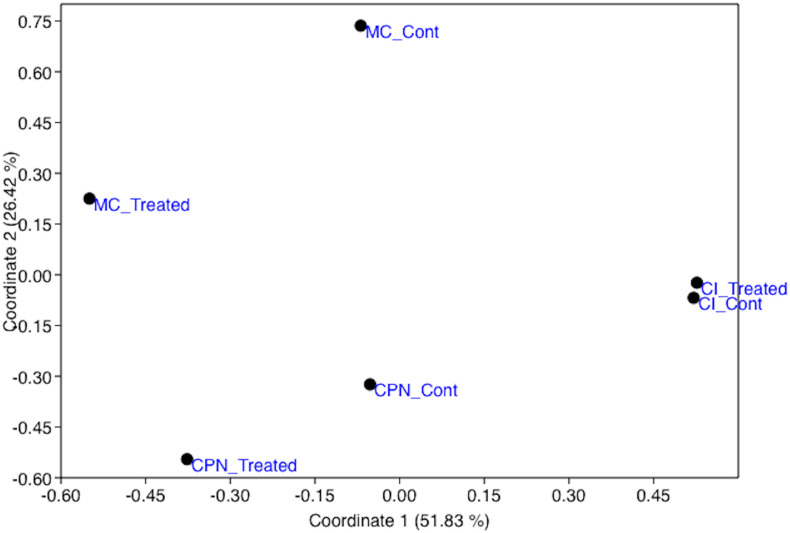
Principal coordinates analysis (PCoA) showing the relationship between the bacterial population structures of the stone and plaster samples in the Maya archeological site, untreated and treated with the M-3P nutritional solution (based on Bray–Curtis index). CI: Temple structure (Structure 18); CPN: stone block treated in the LACEM laboratory; and MC: lime plaster block treated in the LACEM laboratory.

Beta-diversity revealed significant differences in bacterial community structure and abundance between the studied samples before and after the M-3P treatment. The samples formed three distinct clusters, in accordance with the structure type, localization and substrate type, on the principal coordinate analysis (PCoA = multidimensional scaling, MDS) plot of the bacterial community composition (using OTU abundance) of all tested samples (Figure 2). Consequently, the analysis of the CI, CPN, and MC samples indicated that they were clearly classified according to the type of substrate (mortar or tuff) and treatment application.

### Bacterial Community Composition and Structure

**FIGURE 3:**
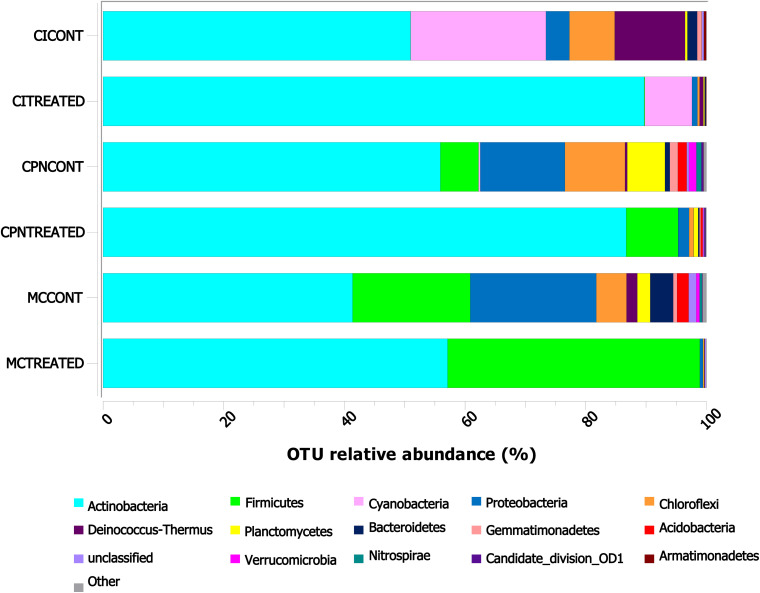
Taxonomic distribution of the bacterial community at phylum level in the stone and plaster samples of the Maya archeological site. Averages of relative abundance of three biological replicates from each sample are represented. Only phyla with a relative abundance of more than 0.1% are represented. CI: stone block of the Temple structure; CPN: stone block treated at the LACEM laboratory; and MC: lime plaster block treated at the LACEM laboratory.

Among the 30 phyla determined in all three samples, *Actinobacteria*, *Firmicutes*, *Cyanobacteria*, *Proteobacteria*, *Chloroflexi, Deinococcus-Thermus*, *and Planctomycetes* were abundantly identified by >1% of the total communities (Figure 3). Some other phyla such as *Bacteroidetes*, *Gemmatimonadetes*, *Acidobacteria*, *Verrumicrobia*, *Nitrospirae*, Candidate_division_OD1, *Armatimonadetes*, and Candidate_division_TM7 were detected in proportions below 1%.

In the stone sample treated *in situ* (CI), many phyla including *Cyanobacteria*, *Deinococcus-Thermus*, *Chloroflexi*, *Proteobacteria*, *Bacteroidetes*, and *Gemmatimonadetes* reduced their proportions from 22.43, 11.68, 7.49, 3.90, 1.63, and 0.76% to 7.83, 0.64, 0.35, 0.91, 0.20, and 0.01% after treatment with the M-3P nutritional solution, respectively, while *Actinobacteria* increased from 50.96% to 89.68% after the treatment (Figure 3). In the laboratory-treated stone sample (CPN), *Actinobacteria* and *Firmicutes* increased from 55.90 and 6.33% to 86.71 and 8.62%, respectively, after treatment application. While others like *Proteobacteria*, *Chloroflexi*, *Planctomycetes*, *Acidobacteria*, *Gemmatimonadetes*, *Verrumicrobia*, *Nitrospirae*, and *Bacteroidetes* significantly reduced their proportions from 14.03, 9.95, 6.24, 1.46, 1.34, 1.24, 0.83, and 0.80% to 1.81, 0.71, 0.75, 0.35, 0.20, 0.13, 0.07, and 0.22%, respectively, after the bioconsolidation treatment. In the lime plaster sample (MC), *Actinobacteria* and *Firmicutes* increased their relative abundance after the treatment from 41.37 and 19.49% to 57.11, and 41.81%, respectively. In contrast, phyla such as *Proteobacteria*, *Chloroflexi*, *Bacteroidetes*, *Planctomycetes*, *Acidobacteria*, and *Deinococcus*-*Thermus* reduced their relative abundance from 20.91, 4.99, 3.81, 2.14, 1.92, and 1.79% to 0.52, 0.12, 0.06, 0.06, 0.04, and 0.00% after the treatment.

**FIGURE 4:**
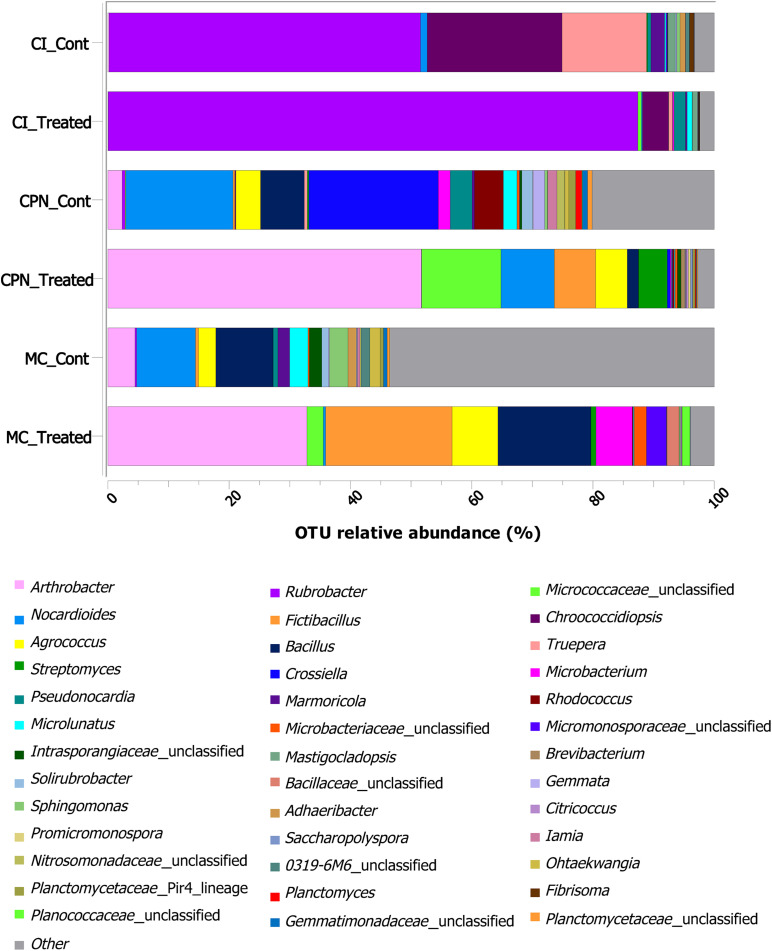
Taxonomic distribution of the bacterial community at genus level in the stone and plaster samples of the Maya archeological site. Averages of relative abundance of three biological replicates from each sample are represented. Only genera with a relative abundance of more than 0.2% are represented. CI: stone block of the Temple structure; CPN: stone block treated at the LACEM laboratory; and MC: lime plaster block treated at the LACEM laboratory.

At genus level, identification of the bacterial groups revealed the presence of 485 different genera in the bacterial communities of all samples (Figure 4 and Supplementary Table 1). After the application of the *in situ* treatment (sample CI), genera such as *Rubrobacter, Pseudonocardia, Microlunatus, Pseudomonas, Propioniferax, Microbacterium, Friedmanniella*, and unclassified-*Micrococcaceae*, among others, increased their proportion in comparison with untreated samples (i.e., samples collected before treatment), while others like *Chroococcidiopsis, Mastigocladopsis, Truepera, Marmoricola, Nocardioides, Fibrisoma*, and unclassified-*Micromonosporaceae*, decreased their proportions, by more than 50% in some cases, after the M-3P treatment. The untreated laboratory stone (CPN_CONT) was colonized by many bacteria including *Crossiella, Nocardioides, Bacillus, Rhodococcus, Agrococcus, Pseudonocardia, Arthrobacter, Microlunatus, Microbacterium, Gemmata, Solirubrobacter, Iamia*, unclassified-*Nitrosomonadaceae*, unclassified-*Planctomycetaceae, Nitrospira*, and *Planctomyces*. All these bacteria reduced their relative abundance after the treatment with the M-3P nutritional solution, with the exception of *Streptomyces*, unclassified-*Micrococcaceae*, *Fictibacillus*, *Agrococcus* and *Arthrobacter*, which increased from 0.25 to 4.75%, from 0.11 to 13.08%, from 0.3 to 6.82%, from 4.09 to 5.19%, and from 2.36 to 51.73%, respectively. A more diverse community was observed in the lime plaster sample (MC sample), where genera such as *Nocardioides, Bacillus, Oxalophagus, Arthrobacter, Propionibacterium, Lachnospiraceae_Incertae_Sedis*, unclassified-*Rhizobiales, Sphin- gomonas, Microlunatus, Agrococcus*, unclassified-*Acetobacteraceae*, unclassified-*Intrasporangiaceae, Marmoricola*, unclassified-*Paenibacillaceae, Deinococcus, Microvirga, Othaekwangia, Blastococcus, Gaiella, Adheribacter, Solirubrobacter, Acinetobacter*, and *Granulicella* dominated the bacterial community of the lime plaster before treatment. After the M-3P bioconsolidation treatment these bacteria significantly reduced their proportions. Other genera increased their relative abundance after the treatment such as *Arthrobacter*, *Fictibacillus*, *Bacillus*, and *Agrococcus*, which increased their relative abundance from 4.47, 0.5, 9.45, and 2.85 to 32.83, 20.86, 15.31, and 7.59%, respectively. *Microbacterium*, *Streptomyces*, *Paenibacillus*, and unclassified members of *Micromonosporaceae*, *Micrococcaceae*, *Microbacteriaceae*, *Bacillaceae*, and *Planococcaceae*, also increased their relative abundance after the application of the treatment in MC sample and passed from less than 0.5% to 5.99, 0.84, 0.84, 3.30, 2.69, 2.04, 1.96, and 1.29%, respectively.

### Statistical Analyses and Correlation Network

**FIGURE 5:**
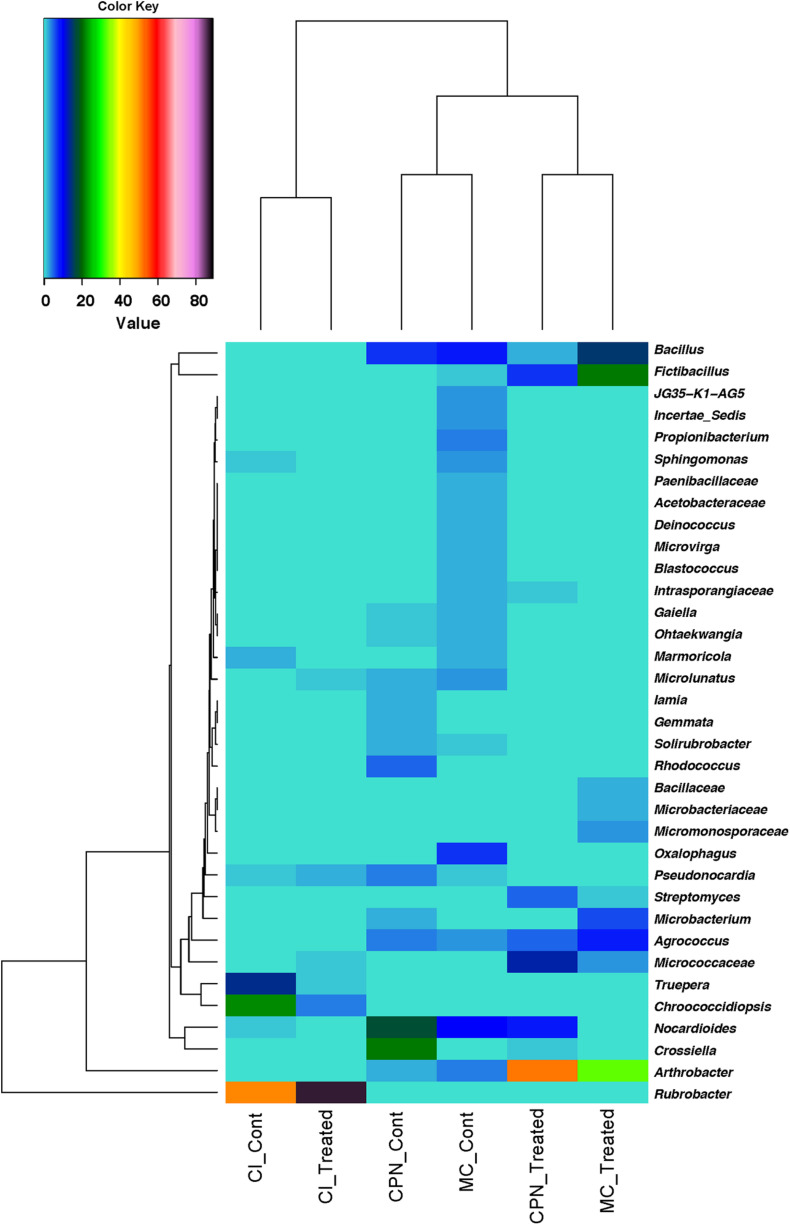
Heatmap based on the relative abundance of genera with an average abundance of >1.5% in at least one sample/replicate. Averages of relative abundance of three biological replicates from each sample are represented.

To further identify the similarity in abundance among the bacterial communities, a heatmap was produced based on the relative abundance of the genera with an average abundance of >1.5% in at least one sample, which were defined as dominant. Abundance of 35 major genera present in all the samples was illustrated (Figure 5). Major genera with occupancies of >80% of all samples were defined as common genera in the present study. Among these 35 major genera, 20 belonging mainly to *Actinobacteria*, 6 to *Firmicutes*, 4 to *Proteobacteria*, and 2 to *Deinococcus-Thermus* were considered as common genera. These included *Rubrobacter*, *Arthrobacter*, *Crossiella*, *Fictibacillus*, *Nocardioides*, *Bacillus*, *Truepera*, *Micrococcaceae*, *Agrococcus*, *Oxalophagus*, *Microbacterium*, *Rhodococcus*, *Streptomyces*, *Propionibacterium*, and *Pseudonocardia*.

Similarity of percentages analysis (SIMPER) was used to determine the relative contribution of each individual taxon to the dissimilarity among the three substrates (CI, CPN, and MC). The average Bray–Curtis dissimilarity and the contribution of each genus to the total dissimilarity between communities of treated and untreated samples were calculated, and the top major genera responsible for the microbial community difference (>98% contribution to cumulative dissimilarity) are summarized in Tables 2, 3. Among the genera responsible for dissimilarity in the communities of untreated samples, *Rubrobacter* had the largest dissimilarity contribution (21.33%), followed by *Chroococcidiopsis* (9.27%), *Crossiella* (7.13%), *Nocardioides* (6.24%), *Truepera* (5.83%), *Bacillus* (4.09%), *Oxalophagus* (2.78%), *Arthrobacter* (1.78%), *Agrococcus* (1.66%), *Propionibacterium* (1.62%), *Rhodococcus* (1.58%), *Microlunatus* (1.53%), *Sphingomonas* (1.15%), and *Pseudonocardia* (1.10%). After the bio-consolidation treatment, the genera contributing to the dissimilarity between the samples changed to some extent, being *Rubrobacter* again the largest dissimilarity contributor followed by *Arthrobacter* (21.16%), *Fictibacillus* (8.53%), *Bacillus* (6.26%), unclassified_*Micrococcaceae* (5.11%), *Nocardioides* (3.54%), *Agrococcus* (3.10%), *Microbacterium* (2.39%), *Streptomyces* (1.93%), *Chroococcidiopsis* (1.76%), and unclassified_*Micromonosporaceae* (1.35%).

**TABLE 2:** SIMPER analysis of bacterial community dissimilarity (>98% of contribution to cumulative dissimilarity) of the three untreated Maya substrates (CI_CONT: stone sample *in situ*; CPN_CONT: stone sample in the LACEM laboratory; and MC_CONT: lime plaster in the LACEM laboratory).

Taxon	Avg dissimilarity (%)	Contribution to dissimilarity (%)	Cumulative dissimilarity (%)	Mean abundance (%)		
				
				Mean CI_CONT	Mean CPN_CONT	Mean MC_CONT
*Rubrobacter*	18.24	21.33	21.33	51.3	0.494	0.282
*Chroococcidiopsis*	7.931	9.276	30.61	22.2	0.153	0
*Crossiella*	6.092	7.125	37.73	0.00152	21.3	0
*Nocardioides*	5.333	6.236	43.97	1.1	17.6	9.63
*Truepera*	4.981	5.826	49.79	13.9	0.497	0
*Bacillus*	3.496	4.088	53.88	0.00442	7.18	9.4
*Oxalophagus*	2.374	2.776	56.66	0	0.00134	6.65
*Arthrobacter*	1.519	1.776	58.43	0.198	2.35	4.45
*Agrococcus*	1.421	1.662	60.09	0.00656	4.09	2.84
*Propionibacterium*	1.381	1.615	61.71	0.00694	0.0196	3.87
*Rhodococcus*	1.354	1.583	63.29	0.000829	4.74	0
*Microlunatus*	1.31	1.532	64.82	0.22	2.25	3.02
*Incertae_Sedis*	1.178	1.377	66.2	0	0	3.3
Unclassified_JG35-K1-AG5	1.133	1.325	67.53	0	0	3.17
*Sphingomonas*	0.9831	1.15	68.68	0.512	0.493	3.06
*Pseudonocardia*	0.9415	1.101	69.78	0.54	3.58	0.761
Unclassified_*Acetobacteraceae*	0.8125	0.9502	70.73	0.0348	0.0135	2.27
Unclassified_*Intrasporangiaceae*	0.6897	0.8066	71.53	0.118	0.312	2.05
*Deinococcus*	0.6463	0.7558	72.29	0.101	0	1.75
Unclassified_*Paenibacillaceae*	0.6423	0.7512	73.04	0	0.00293	1.8
*Ohtaekwangia*	0.6238	0.7295	73.77	0	0.619	1.68
*Microvirga*	0.6036	0.7059	74.48	0.00809	0.0369	1.7
*Marmoricola*	0.6028	0.705	75.18	2.24	0.415	1.93
*Gaiella*	0.5787	0.6768	75.86	0	0.809	1.62
*Blastococcus*	0.5766	0.6744	76.53	0.0258	0.0181	1.63
*Microbacterium*	0.5615	0.6567	77.19	0.0143	1.96	0
*Gemmata*	0.5386	0.6299	77.82	0.106	1.91	0.184
*Solirubrobacter*	0.5352	0.6259	78.45	0.165	1.81	1.06
*Iamia*	0.4541	0.5311	78.98	0.0403	1.53	0.424

**TABLE 3:** SIMPER analysis of bacterial community dissimilarity (>98% of contribution to cumulative dissimilarity) of the three treated Maya substrates (CI_TREAT: stone sample treated *in situ*; CPN_TREAT: stone sample treated in the LACEM laboratory; and MC_TREAT: lime plaster treated in the LACEM laboratory).

Taxon	Avg dissimilarity (%)	Contribution to dissimilarity (%)	Cumulative dissimilarity (%)	Mean abundance (%)		
				
				Mean CI_Treat	Mean CPN_Treat	Mean MC_Treat
*Rubrobacter*	29.09	35.74	35.74	87.30	0.02	0.01
*Arthrobacter*	17.22	21.16	56.90	0.06	51.70	32.80
*Fictibacillus*	6.94	8.53	65.43	0.01	6.82	20.80
*Bacillus*	5.09	6.26	71.69	0.01	1.84	15.30
Unclassified_*Micrococcaceae*	4.16	5.11	76.80	0.59	13.10	2.69
*Nocardioides*	2.88	3.54	80.35	0.15	8.80	0.37
*Agrococcus*	2.52	3.10	83.44	0.01	5.19	7.58
*Microbacterium*	1.94	2.39	85.83	0.22	0.20	5.98
*Streptomyces*	1.57	1.93	87.76	0.02	4.73	0.84
*Chroococcidiopsis*	1.43	1.76	89.52	4.30	0.00	0.00
Unclassified_*Micromonosporaceae*	1.10	1.35	90.87	0.02	0.02	3.30
Unclassified_*Microbacteriaceae*	0.68	0.83	91.70	0.01	0.36	2.03
Unclassified_*Bacillaceae*	0.65	0.80	92.50	0.00	0.08	1.96
*Pseudonocardia*	0.62	0.77	93.27	1.89	0.13	0.03

**FIGURE 6:**
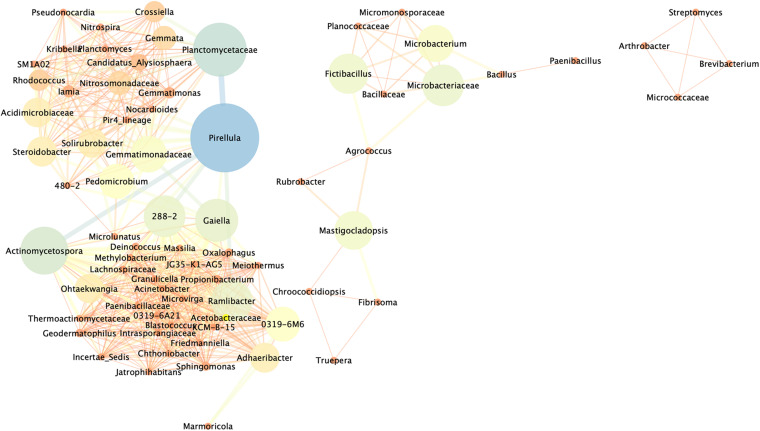
Network analysis revealing co-occurrence patterns among bacterial taxa in the different samples of the Maya archeological site. The nodes and edges are colored according to Betweenness Centrality. Only taxa with a relative abundance of more than 0.5% in at least one sample were used. Each connection represents strong correlations based on Pearson’s correlation coefficient (ρ of >0.8 and ρ of > –0.8). The thickness of each line is proportional to the significance of the interaction (ρ-value), and the size of the circle (the node) is proportional to the number of connections, i.e., the degree, of bacterial genera.

To comprehensively understand the interaction effects between bacteria detected before and after the application of the nutritional solution, a network of significant co-occurrence and co-exclusion relationships among genera was constructed on the basis of strong Pearson correlation matrix (ρ > 0.8 or ρ < −0.8) (Figure 6). Among the 485 bacterial genera detected, those showing a relative abundance of more than 0.5% in at least one sample were used to construct this association network. A total of 73 nodes and 699 edges were identified. The network could be divided into three clear clusters formed by nodes interacting more strongly among themselves than with others. The three clusters were occupied by 4, 14, and 55 nodes out of the 73 total vertices and, with the exception of three (i.e., *Rubrobacter*, *Agrococcus*, and *Mastigocladopsis*) all were found to be strongly positively correlated. In the first cluster, *Arthrobacter*, *Streptomyces*, *Micrococcaceae*, and *Brevibacterium* were equally distributed and no keystones were distinguished over the rest of the bacteria, being nodes with low mean degree and low Betweenness Centrality. *Microbacterium*, *Fictibacillus*, *Microbacteriaceae*, and *Mastigocladopsis* were considered potential keystones in the second cluster showing strong correlation connections with the rest of core bacteria in the cluster. In the most complex cluster, the 55 nodes formed a complex network with strong interactions and taxa like *Planctomycetaceae*, *Pirellula*, *Gaiella*, *Solirubrobacter*, *Gemmatimonadaceae*, *Pedomicrobium*, *Acidimicrobiaceae*, *Ramlibacter*, among others, which were considered keystone bacteria, exhibiting strong positive correlations with other core genera. Finally, only three genera *Rubrobacter*, *Agrococcus* and *Mastigocladopsis* presented negative Pearson correlation matrix (ρ < −0.8) in the community, meaning that the presence of *Rubrobacter* was negatively correlated with the presence of *Agrococcus* and *Mastigocladopsis* in the bacterial population. The presence of *Matigocladopsis* was also negatively correlated with the presence of *Agrococcus*. For this reason, *Rubrobacter* was only present in the temple Structure 18, whereas *Agrococcus* was only detected in the stone sample CPN and the mortar sample MC treated indoors in the laboratory.

## Discussion

### Before Treatment

High-throughput sequencing results in our study revealed a rich diversity of bacterial communities on the deteriorated stone and plaster samples of the Maya archeological site. We identified a total of 161 genera in the CI_Cont, 309 in CPN_Cont, and 141 in MC_Cont. Association networks constructed to the genus level showed mainly positive correlation patterns with strong and complex connections, as well as some negative correlations with loose and simple connections. These interactions between microbial taxa in stone and plaster artworks indicated significant co-occurrence patterns helping to decipher the relationships between community members. Most of these genera belong to *Actinobacteria*, *Cyanobacteria*, and *Firmicutes*, which are identified as the keystone taxa and contribute to the association of the bacterial community, suggesting either shared or preferred environmental conditions or the performance of similar or complementary functions (Jroundi et al., 2020).

Generally, microbial communities in archeological sites have been reported to have distinct diversity patterns under different environmental conditions (Gaylarde et al., 2007; Piñar et al., 2015; Li et al., 2016; Dhami et al., 2017; Mihajlovski et al., 2017; Sterflinger et al., 2018). The results of this study show that the microbial community patterns detected on archeological/historic stone and mortar surfaces not only depended on the overall environmental conditions, but were also greatly influenced by the location of each individual building or decorative element within the archeological Maya site. Data presented (in Figure 4 and Table 2) reveal that the microbial population of the untreated stone sample (CI) from a vertical wall of Structure 18 was dominated by *Rubrobacter*, *Chroococcidiopsis*, and *Truepera*. Remarkably, these bacteria were completely absent from the remaining untreated stone and plaster floor samples (CPN and MC), which were in direct contact with the ground. Consequently, these genera, and particularly *Rubrobacter*, had the largest contribution to bacterial community dissimilarity. Members of *Rubrobacter* sp. have been isolated from a range of environmental samples including different soil niches (Holmes et al., 2000) and many monuments including the Mayan ruins of Uxmal (Mexico) (Ortega-Morales et al., 2004; Sterflinger and Piñar, 2013; Mihajlovski et al., 2017; Adamiak et al., 2018). They are known to be thermophilic, halotolerant, and gamma-radiation resistant bacteria, which also colonize salt laden building materials (Laiz et al., 2009; Jurado et al., 2012; Adamiak et al., 2018). The preferential colonization of *Rubrobacter* on the vertical walls of Structure 18 confirms previous results, indicating that their resistance toward temporary desiccation and intensive daylight irradiation would bestow them a selective advantage over other bacteria (Laiz et al., 2009). Often, the presence of *Rubrobacter* has been associated with biodeterioration processes/phenomena in monuments, including pink discoloration (Ortega-Morales et al., 2004; Laiz et al., 2009; Sterflinger and Piñar, 2013; Mihajlovski et al., 2017; Adamiak et al., 2018). Laiz et al. (2009) associated stone biodeterioration with the *Rubrobacter*-induced precipitation of struvite (NH_4_MgPO_4_⋅6H_2_O), a mineral which is also produced by other carbonatogenic bacteria such as *Myxococcus xanthus* cultured in Mg- and phosphate-containing medium (González-Muñoz et al., 2008). Note that the latter bacterium has an outstanding biomineralization capacity and is known to produce cementing calcium carbonate under adequate conditions (Rodriguez-Navarro et al., 2003). Furthermore, the possible role of struvite precipitation by *Rubrobacter* inducing salt damage in stone as suggested by Laiz et al. (2009) is questionable, considering its low solubility. Struvite is a sparingly soluble phase with solubility of 4.07 × 10^–5^ mol/L at 25°C (Hanhoun et al., 2011), a value slightly lower than that of calcite (5.75 × 10^–5^ mol/L), a benign sparingly soluble carbonate. In fact, calcite effectively cements porous stone, being the basis of the bacterial biomineralization consolidation treatment (Rodriguez-Navarro et al., 2015). It seems even possible that struvite could act as a consolidant as it has been reported in the case of other alkaline-earth phosphates such as hydroxyapatite (Sassoni et al., 2011).

In general, the microbial communities living in the archeological sites or historical buildings all over the world are linked to environmental conditions (Gaylarde et al., 2007; Li et al., 2016). The high-throughput sequencing results in our study revealed a rich diversity of bacterial communities on the stone and plaster samples of the Maya archeological site. Indeed, although samples showed similar signs of biodeterioration and microbial colonization, overall, the microbial diversity in this study varied enormously in composition, seemingly affected by sample location, substrate type (i.e., silicate tuff stone *vs.* calcium carbonate lime plaster), and environmental conditions.

The diversity of the bacterial communities in samples CPN (stone) and MC (mortar) was almost twice as large as compared with sample CI (stone in Structure 18). Possibly, the direct contact with the soil microbiota of the ground had a significant impact on the bacterial population in the former samples. Remarkably, the degree of similarity was higher between the stone sample CPN and mortar sample MC than between the two volcanic tuff stone samples, indicating that the original sample location was of greater importance than substrate characteristics (composition, mineralogy and textural features). Additionally, *Bacillus* was detected in all samples, which was not surprising since many members of these bacteria have been identified on stone surfaces and are frequently used for the bioconsolidation of deteriorated historical monuments and sculptures of different substrate types including calcareous stone, concrete and mortars (Dhami et al., 2012, 2014; Achal et al., 2013; Wang et al., 2014; Montaño-Salazar et al., 2018). Other genera such as *Agrococcus*, *Pseudonocardia, Microlunatus, Pseudomonas, Propioniferax, Microbacterium*, and *Friedmanniella* (the latter 4 were present at concentrations below 0.1% and are not included in Figure 4) were also detected in our study, which have the ability to survive in extreme environments and were previously identified in archeological sites (Li et al., 2016), caves (Portillo et al., 2009; Fang et al., 2017), Roman catacombs (Krakova et al., 2015), wall paintings of a medieval chapel in Austria (Schabereiter-Gurtner et al., 2001), and on the tomb walls of an Etruscan Necropolis (Diaz-Herraiz et al., 2013). Although present at low relative abundance, some bacteria are able to play an important role in many environments because of their high metabolic activity. Some of them such as *Pseudomonas* strains have been shown to precipitate calcium carbonate crystals in the surface of bacterial isolates and are considered among the most common microorganisms detected on stone monuments (Li et al., 2018). *Pseudonocardia* sp. have been previously detected as a dominant component of the microbial community in caves, wall paintings, tomb walls and Roman catacombs (Portillo et al., 2009; Diaz-Herraiz et al., 2013; Krakova et al., 2015). Some studies demonstrated that members of this genus, and of the phylum *Actinobacteria* in general, were able to precipitate calcium carbonate in culture media supplemented by different calcium salts (Fang et al., 2017), suggesting their potential to precipitate such calcium carbonate to consolidate stone of cultural heritage; although this needs to be confirmed by further studies.

Finally, members of the phylum *Acidobacteria* were detected in CPN and MC samples. Many studies have reported the presence of these non-culturable *Acidobacteria* in cave habitats, including catacombs (Zimmermann et al., 2006). *Acidobacteria* seem to adapt well to low nutrient substrates, which corroborates their possible oligotrophic lifestyle in many environmental habitats (Naether et al., 2012). They are also known to be acidophilic, strictly aerobic, chemo-organotrophic bacteria, capable of degrading sugars and some polysaccharides (Pankratov et al., 2012).

### After Treatment

Regardless of treatment location (i.e., *in situ* or *ex situ*) the application of the patented nutritional solution (M-3P) caused an important change in bacterial population in all samples, particularly increasing the amount of *Actinobacteria* in samples CI, CPN, and MC by 89.68, 86.71, and 57.11%, respectively. The increase in the amount of carbonatogenic *Actinobacteria* can be considered as an indicator for the potential efficacy of this bioconsolidation treatment since this bacterial phylum is known to be able to utilize various carbon and nitrogen sources and produce calcium carbonate (Fang et al., 2017). Remarkably, the increase in *Actinobacteria* was more pronounced in stone samples as compared to the mortar sample, where *Firmicutes* (dominated by the genera *Fictibacillus* and *Bacillus*) became the second most dominant phylum (increasing from 19.49 to 41.81%). These bacteria are found in various environments and can survive extreme conditions. Some of them are known for their endospores production, which makes them resistant to desiccation (Parkes and Sass, 2009; Nazir et al., 2019). These bacteria, specially *Bacillus* spp., have been suggested to have a direct relationship with carbonate deposition and have been widely used for the consolidation and strengthening of sand columns and soils, the repairment of concrete cracks and in the field of limestone building conservation, showing in all cases a high capacity to precipitate calcite and vaterite (Le Métayer-Levrel et al., 1999; De Muynck et al., 2010, 2013; García et al., 2016).

Our results show that the composition of the original bacterial population has an important influence on the bacterial population developed after the consolidation treatment. In the case of the stone sample from Structure 18 (CI), *Rubrobacter* remained the dominant genus, experiencing an increase in relative abundance of 66% after the bioconsolidation treatment, whereas all other genera suffered an, in certain cases dramatic (i.e., *Truepera* and *Chroococcidiopsis*), decrease. It is very likely that *Rubrobacter* would play a relevant role in the production of calcium carbonate cement, without any detrimental side effects (e.g., salt damage or discoloration). As stated above, it is very unlikely that *Rubrobacter*-induced struvite precipitation could have any salt damaging effect. Besides, struvite formation after application of the M-3P treatment is improbable, as this medium lacks magnesium. In contrast, it is very likely that *Rubrobacter* can effectively contribute to the biomineralization of calcium carbonates. On the one hand, it has been shown that *Rubrobacter* spp. are associated with CaCO_3_ precipitation in endostromatolites (Pellerin et al., 2009). On the other hand, the observed increase in *Rubrobacter* abundance after the M-3P treatment is consistent with this bacterium being able to use amino acids for its metabolic activity. It is thus expected that it contributes to an alkalinization of the medium resulting in the formation of cementing calcium carbonates. Regarding any potential problems of discoloration associated with this bacterium, as it is indicated in the Materials and Methods section, we covered the substrates during the whole duration of the treatment to prevent light irradiation of treated surfaces, precisely to avoid any possible pigmentation by bacteria. Actually, we observed no color changes/discoloration 3 months after treatment application. In any case, a detailed characterization of the untreated and treated substrate (i.e., compositional, textural and physico-mechanical analysis) is currently underway in order to proof bacterial carbonate biomineralization and to rule out possible negative side effects.

In samples CPN and MC, *Arthrobacter* showed the strongest response to the nutritional supply and became the dominant genus, augmenting its relative abundance by a factor of 21.9x and 7.3x, respectively. Consequently, they have, after *Rubrobacter*, the second largest contribution to bacterial community dissimilarity in treated samples. Jroundi et al. (2012) have shown that *Arthrobacter* species are promising candidates for successful biomineralization induced by the application of a nutritional solution that selectively activates carbonatogenic bacteria. The observed improved mechanical strength in treated monumental stone and mortars, especially in the case of *Arthrobacter crystallopoietes*, has even prompted their consideration for industrial applications (Park et al., 2010; Dhami et al., 2012, 2014; Jroundi et al., 2012; Montaño-Salazar et al., 2018). Other carbonatogenic bacteria, including *Fictibacillus*, *Bacillus*, *Streptomyces*, and *Agrococcus* also reacted positively to the treatment and increased their abundance.

Remarkably, members of the phylum *Acidobacteria* were also significantly affected by the bioconsolidation treatment, experiencing an important decrease of 76 and 98% in abundance in the case of sample CPN and MC, respectively. Zimmermann et al. (2006) reported on their active negative role on cultural heritage in collaboration with other microorganisms. The acidic metabolic products of these bacteria have been shown to cause severe irreversible damage to stone monuments, leading to the dissolution of the stone matrix, especially in the case of calcareous rocks (Douglas and Beveridge, 1998; Warscheid and Braams, 2000). Therefore, such a negative impact of this bioconsolidation treatment on *Acidobacteria* can be taken as additional evidence for the suitability of the proposed strategy for the consolidation and conservation of monuments and archeological sites in tropical hot and humid environments.

## Conclusion

In this study, we showed for the first time the enormous impact of the bioconsolidation methodology based on the application of a sterile nutritive solution (M-3P) on the indigenous bacteria present in stone and plaster at the Maya archeological site of Copan. A detailed characterization of the bacterial population evolution revealed that the bioconsolidation treatment induced a significant increase in beneficial indigenous carbonatogenic bacteria and a concomitant suppression of potentially damaging *Acidobacteria*. Positive results were obtained under varying treatment conditions (i.e., treatments in the laboratory and *in situ*) and independent of substrate type (i.e., stone and mortar). This study also revealed that the original bacterial population of the substrate was decisive for the population evolution upon treatment, being influenced by the location of the sample within the archeological site (i.e., stone samples from vertical walls versus semi-buried stone and floor plaster fragments with contact to soil microbiota). Importantly, the application of the sterile M-3P nutritive solution did not result in the flourishing of any damaging bacteria in this hot and humid environment. At present we are evaluating the consolidation effect of such a treatment applied on Maya stones and plasters of Copan, focusing on the quantification and distribution of the newly formed bacterial calcium carbonate cement and its impact on the treated substrates’ physical-mechanical properties. Combined results will lay the basis for the development of an environmentally friendly compatible conservation treatment for monuments in hot and humid climates based on the bacterial precipitation of cementing calcium carbonate for the consolidation of plaster and stone.

Furthermore, the relative safety and ease of application of the treatment proposed here as compared to conventional biotreatments using bacteria inoculums are worth highlighting. The former simply consists of spraying the patented nutritional solution onto the degraded substrate surface, allowing for large-scale applications without posing any significant risk to operator or environment. Admittedly, the biotreatment is labor intensive (i.e., relatively large number of applications are required). However, overall material-related treatment costs are comparable to those of conventional consolidation treatments.

## Data Availability Statement

All raw sequences used in this study are available in the sequence read archive (SRA) at NCBI database under the SRA accession number PRJNA650538.

## Author Contributions

CR-N and MTG-M conceived the concept and led this project. CR-N and FJ designed the experimental setup. CR-N, KE, and ER-A set up and managed the treatment and the sample collections. FJ performed all laboratory works, analyzed the data, and created the graphs and figures. FJ, KE, and CR-N were major contributors in writing the manuscript with critical input from MTG-M, and ER-A. All authors read and approved the final version of the manuscript.

## Conflict of Interest

The authors declare that the research was conducted in the absence of any commercial or financial relationships that could be construed as a potential conflict of interest.

## Footnotes

1 http://www.lgcgroup.com/

2 http://bioware.soton.ac.uk
